# Methodology for Selecting the Operating Conditions of a Vibration Generator Used in the Hot-Dip Galvanizing Process

**DOI:** 10.3390/ma14082042

**Published:** 2021-04-19

**Authors:** Wojciech Kacalak, Igor Maciejewski, Dariusz Lipiński, Błażej Bałasz

**Affiliations:** Faculty of Mechanical Engineering, Koszalin University of Technology, 75-620 Koszalin, Poland; wojciech.kacalak@tu.koszalin.pl (W.K.); igor.maciejewski@tu.koszalin.pl (I.M.); blazej.balasz@tu.koszalin.pl (B.B.)

**Keywords:** vibration, hot-dip galvanizing, generator, simulation, zinc coating

## Abstract

A simulation model and the results of experimental tests of a vibration generator in applications for the hot-dip galvanizing process are presented. The parameters of the work of the asynchronous motor forcing the system vibrations were determined, as well as the degree of unbalance enabling the vibrations of galvanized elements weighing up to 500 kg to be forced. Simulation and experimental tests of the designed and then constructed vibration generator were carried out at different intensities of the unbalanced rotating mass of the motor. Based on the obtained test results, the generator operating conditions were determined at which the highest values of the amplitude of vibrations transmitted through the suspension system to the galvanized elements were obtained.

## 1. Introduction

Galvanizing is a technology for anticorrosive protection of the surface of metal products by applying a mechanically durable zinc coating. One of the most important galvanization technologies is hot-dip galvanizing for anticorrosive protection of the surface of metal products by applying a mechanically durable zinc coating. Zinc coatings can be applied in the following processes: hot-dip galvanizing, electroplating, and zinc spraying. Hot-dip galvanizing makes it possible to obtain good-quality coatings and provides long-term protection [1]. In the conventional hot-dip galvanizing process, the surface of a steel object is cleaned and then immersed in a zinc bath at a temperature of approximately 450 °C [2]. The hot-dip galvanizing process provides a coating with high strength, deformability, and low weight. Research on the hot-dip galvanizing process is mainly related to the composition of the zinc bath and the method of the surface preparation of products [3].

A significant cost of the hot-dip galvanizing process is the cost of zinc. Zinc consumption depends mainly on the thickness of the coating, but is also influenced by specific process conditions resulting in the formation of hard zinc, zinc ashes, oxidation of the bath surface, as well as the formation of solidified zinc droplets [4]. After the hot-dip galvanizing process, the excess of zinc drips from the surface of the steel products by gravity. This results in uneven zinc coating and the accumulation of zinc bath residues in areas that do not allow free zinc flow [5]. As a result, apart from the inaccuracy of the produced zinc coating, the share of waste generated also increases, inter alia, as a result of the mechanical removal of stains and local excessive zinc layers [6].

In the zinc coatings produced on steel elements in the process of hot-dip galvanizing, two layers [7] can be distinguished: the internal diffusion and the external diffusion in the form of a solidifying film during the resurfacing of galvanized elements. The thickness proportions of these layers depend on the parameters of the galvanizing process. The aim is to create a thin diffusion layer with high adhesion and an even but possibly thin outer layer with low porosity. The quality of the produced outer layer depends on the dynamics of zinc displacement on the galvanized product, both during the bath and during the removal of the element from the bath.

The properties of the outer layer can be shaped by the appropriate selection of the speed of the product emerging from the bath (with an estimated speed of about 1.5 m/min [4]). Too slow an extraction speed produces a uniform unalloyed zinc layer. In the case of excessive extraction speed, an uneven zinc layer is formed. The heterogeneity of the zinc layer affects the locally variable corrosion properties of the produced coatings [8]. In hot-dip galvanizing processes, the thickness of the zinc coating can be controlled with the use of air knife wiping [9]. This process removes any excess of liquid zinc with a gas stream. However, the application of this process is limited mainly to the galvanizing processes of products with non-complex shapes (e.g., steel strips) [10].

The formation of the outer layer of zinc on tinned elements is the result of adhesion and cohesion processes described by the equations of motion of incompressible fluids of high viscosity. The formation of the liquid layer depends on the friction forces, dependent on the viscosity, surface tension, and inertia forces [11]. The speed of the zinc bath particles in the boundary layer when the elements are withdrawn from the liquid bath depends on their distance from the surface of the object. On the surface, the speed of the particles is close to the speed of the object, and decreases to zero in the border area of the meniscus. The system of frictional and inertia forces as well as gravity and surface tension shapes the convex meniscus. Increasing the speed of movement of the pulled-out elements increases the lifting forces. This increases the thickness of the liquid zinc layer, which adversely affects the evenness of the layer and increases the consumption of zinc, leading to a reduction in the efficiency of the galvanizing process. The introduction of vibrations increases the forces of dynamic impact on liquid zinc particles, causing the thickness of the layers in zones of excessive thickness and the removal of thin zinc films in the spaces of openings and small-width recesses.

The use of vibration in technological processes has been the subject of many studies [12,13]. For example, in machining, low-amplitude vibrations (from 2 µm to 100 µm) and frequencies ranging from a few Hz to 40 kHz were introduced [12]. Thanks to the appropriate selection of cutting parameters and the parameters of the vibration process, it was possible to reduce the thickness of the cutting layer in [14,15] and to reduce the cutting forces in [16]. As a result, machined surfaces with more-favorable roughness parameters [17] and with higher dimensional accuracy [18] were obtained. In the automated hot-dip galvanizing process of steel sheets, the vibration amplitude of a galvanized element is approximately 1 mm [19]. The uniformity of the thickness of the zinc coating is in this case ensured by the use of air knives. In the case of galvanizing elements of complex shape containing closed profiles, it is not possible to use air knives. The removal of excess zinc can in this case be achieved by inducing low-frequency and low-amplitude vibrations.

The following paper presents a methodology for selecting the operation conditions of a vibration generator that can be used in hot-dip galvanizing processes. The assumption of the modified galvanizing process, in which the stage of removing the products from the zinc bath takes place with the participation of mechanical vibrations with a specific amplitude and frequency, depends on the weight of the suspended galvanized elements. The purpose of the vibrations during the emergence of the elements from the zinc bath is to increase the uniformity of the coating thickness, limiting the formation of thin layers of zinc covering holes with small diameters and shaping a favorable geometric structure of the surface.

The main problem to be solved was the development of the structure of vibration generators and the methodology of selecting parameters for controlling their operation for the assumption that the mass of galvanized elements with their mounting system would be from 500 to 1000 kg. It was assumed that the weight of the casing and other elements suspended on the transport system, to which vibrations should be transmitted to the least extent, would be limited by the parameters of the permissible load of this system. The further part of the work presents the results of experimental tests of the manufactured prototype of a vibration generator for use in the galvanizing process.

## 2. Simulation Model of the Vibration Generator

**Figure 1:**
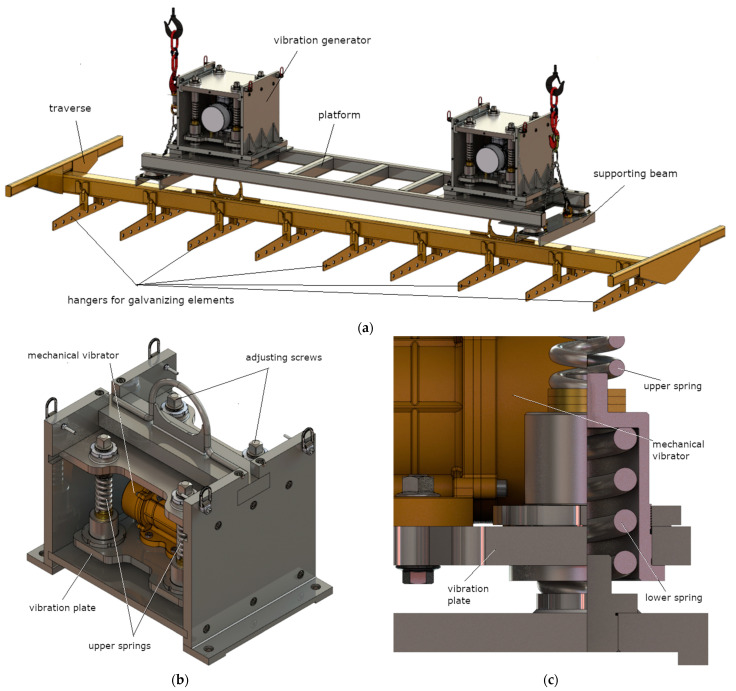
Visualization of the designed vibration system for galvanizing processes (**a**); vibration generator 800 mm (L) × 600 mm (W) × 530 mm (H) (**b**); enlargement of a part of the suspension system (**c**).

The designed mechanical vibration generator for hot-dip galvanization applications is presented in Figure 1. The system includes two vibration generators placed between the gantry and the platform with galvanized items.

The system is forced to vibrate by a mechanical vibrator in which unbalanced masses are set in rotation around the axis of the electric motor. The system vibrations are transferred to the suspended elements for galvanizing through a vibrating plate placed in the spring system. The weight of the galvanized products suspended from the vibrating plate affects the behavior of the entire system.

**Figure 2:**
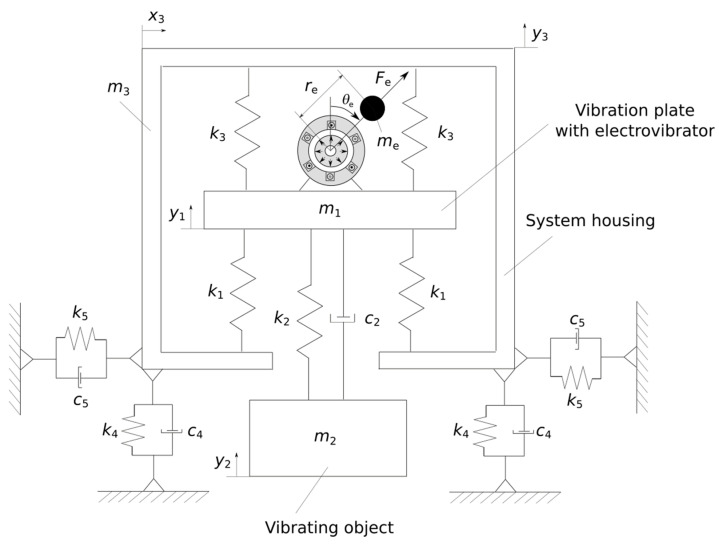
Physical model of the mechanical vibration generator with unbalance of the motor rotating mass.

A physical model of the vibration generator tested in laboratory conditions is presented in Figure 2. The presented system comprises a vibration plate with electric shaker (mass *m_1_*, its vertical movement *y_1_*), the vibrating object (mass *m_2_*, its vertical movement *y_2_*), and the system housing (mass *m_3_*, its vertical movement *y_3_*). The generator housing is mounted using viscoelastic elements, which represent the system support for a specially prepared frame structure. In the case of the presented model, apart from the vertical vibration of individual elements of the generator, the entire system can generate oscillatory movements in the direction labeled as *x_3_*.

Equations of motion for individual system components in the vertical direction are presented in the form of ordinary differential equations, which are presented according to the following relationships:

(1) $$\begin{array}{l} m_{1}{ÿ}_{1}=-4k_{1}(y_{1}-y_{3})-c_{2}({\dot{y}}_{1}-{\dot{y}}_{2})-k_{2}(y_{1}-y_{2})+4k_{3}(y_{3}-y_{1})+F_{e}\cos\theta_{e}\\ m_{2}{ÿ}_{2}=c_{2}({\dot{y}}_{1}-{\dot{y}}_{2})+k_{2}(y_{1}-y_{2})\\ m_{3}{ÿ}_{3}=4k_{1}(y_{1}-y_{3})-4k_{3}(y_{3}-y_{1})-4k_{4}y_{3}-4c_{4}{\dot{y}}_{3} \end{array}$$

where *k_1_* is the stiffness of the lower springs (4 pcs.), *c_2_* and *k_2_* are the damping and stiffness of the rod fixing the element excited to mechanical vibrations, *k_3_* is the stiffness of the upper springs (4 pcs.), *c_4_* and *k_4_* are the damping and stiffness representing the system support, *F_e_* is the centrifugal force of the vibration shaker, and *θ_e_* is the rotational angle of the electric motor.

Vibrations generated by the entire mechanical system in the horizontal direction are explained by using only one ordinary differential equation, as follows:

(2) $$(m_{1}+m_{2}+m_{3}){\overset{..}{x}}_{3}=-4k_{5}x_{3}-4c_{5}{\dot{x}}_{3}+F_{e}\sin\theta$$

where *m*_1_ + *m*_2_ + *m*_3_ is the total mass of the examined vibration generator and *x_3_* is its displacement measured in the horizontal direction.

The centrifugal force of the element introducing both vertical and horizontal vibrations into the system is described with the following relationship:

(3) $$F_{e}=\delta_{e}m_{e}{{\dot{\theta }}_{e}}^{2}r_{e},$$

where *δ_e_* is the intensity of an unbalanced rotating mass of the motor, *m_e_* is the mass of the rotating element, ${\dot{\theta }}_{e}$ is the rotational speed of the electric motor, and *r_e_* is the lever arm of the rotating element.

**Figure 3:**
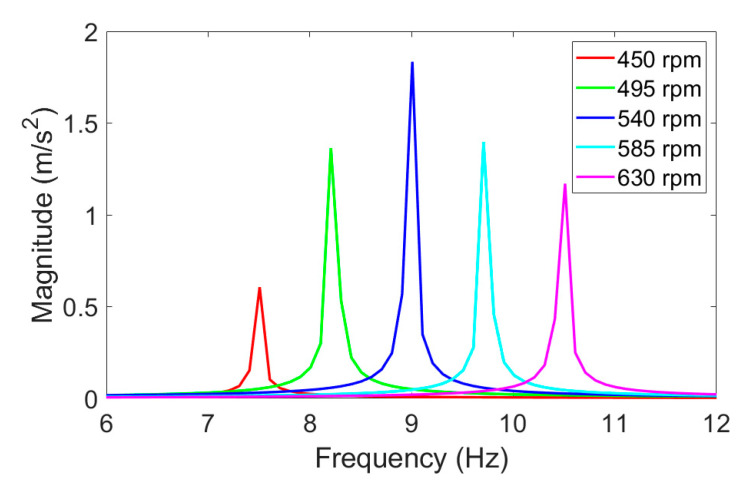
Magnitudes of the vibrating object at different rotational speeds of the electric motor.

By changing the rotational speed of the motor, it was possible to generate vertical vibrations of the system with various vibration intensities. Both the amplitude and the frequency of generated vibrations depended mainly on the harmonically applied force *F_e_* as well as the rotational speed of the electric motor ${\dot{\theta }}_{e}$. Assuming a constant intensity of the rotating mass, maximum magnitudes of the vibrating object were obtained for 540 rpm (Figure 3). This rotational speed caused a damped resonance effect in the mechanical system at a frequency close to 9 Hz. Therefore, further investigations of the proposed vibration generator were performed at its resonance frequency.

## 3. Verification of the Simulation Model in Laboratory Conditions

**Figure 4:**
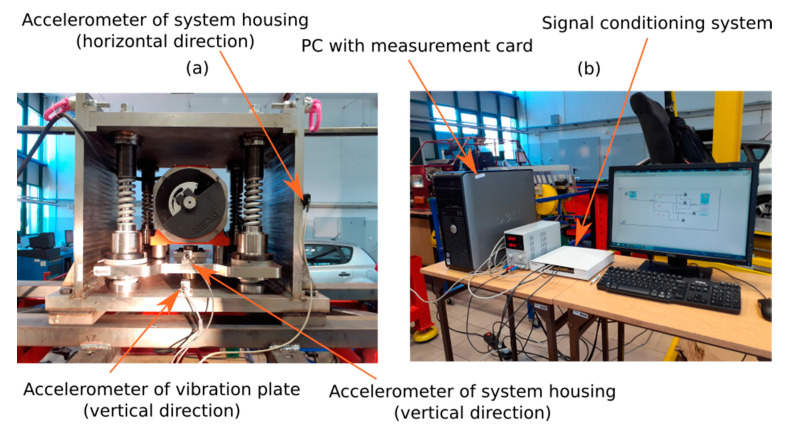
The experimental set-up for the analysis of dynamic behavior of a vibration generator in laboratory conditions (**a**) together with the measurement data acquisition system (**b**).

**Figure 5:**
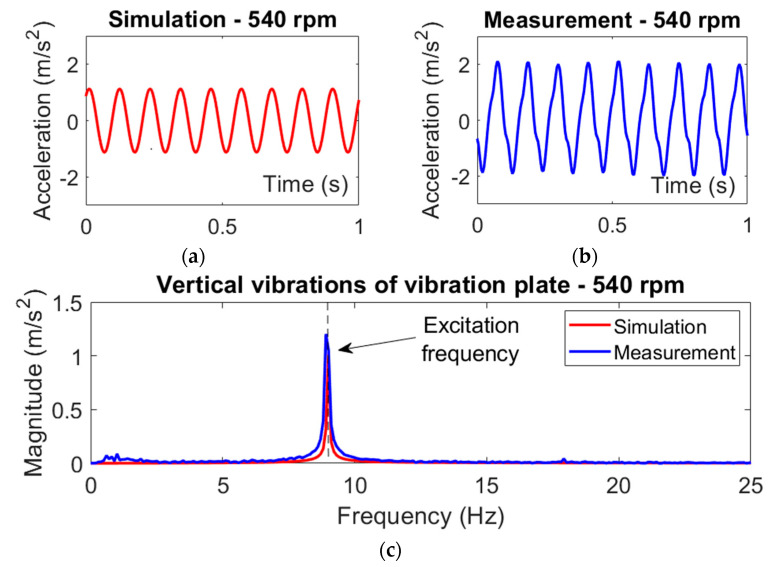
The acceleration waveforms of vertical vibrations of the vibration plate (**a**,**b**) and the corresponding amplitude spectra (**c**) obtained at the rotational speed of 540 rpm and the intensity of rotating mass unbalance 30%.

The experimental setup for the analysis of dynamic behavior of the vibration generator in laboratory conditions is presented in Figure 4. The tested generator was mounted to a specially adapted frame structure and was loaded with a mass of 466 kg (Figure 4a). Three accelerometers were installed (Figure 5b), by which it was possible to measure the vibrations generated on the vibrating plate (connected with the suspension system of galvanized elements) and the vibrations transmitted to the frame structure in the vertical and horizontal directions. In order to measure vertical vibrations of the vibrating platform and the system housing, two piezoelectric accelerometers were employed with a measurement range of ±50 g and a sensitivity equal to 100 mV/g. Successively, one capacitive sensor with a measuring range ±2 g and a sensitivity of 1 V/g was utilized to determine horizontal vibrations of the system housing. PC with the installed National Instruments PCI-6251 measurement card and a compatible signal conditioning system (Figure 4b) was used for data acquisition. The data acquisition rate on each channel was set at 1 kHz, and the acceleration data were recorded during a measurement period of 10 s.

The acceleration signals measured on the test stand described in Figure 4 were filtered in the frequency range of 0.5–25 Hz and were subsequently analyzed by using discrete Fourier transform (DFT). Verification of the simulation model was conducted at an electric motor rotational speed of 540 rpm, which is close to the eigenfrequency of the mechanical structure in the vertical direction of interaction. In this case, the intensity of the unbalanced rotating mass was set at 30%. The parameters of the simulation model are included in Appendix A. Figure 5 presents waveforms of the vertical acceleration of the vibration plate, which were obtained through computer simulation as well as measurements conducted in laboratory conditions. Moreover, the amplitude spectra of the measured vibration acceleration signals were compared for the motor speed of 540 rpm and the selected intensity of rotating mass unbalance.

As shown in Figure 5, a satisfactory agreement of the simulation and measurement results is obtained for the rotational speed of 540 rpm and the unbalance intensity of the rotating mass equal to 30%. Under these operating conditions, the simulated and measured acceleration waveforms of the vibrating plate showed comparable vibration amplitudes. This proves the sufficient reliability of the proposed system model, which is used in this paper to analyze the dynamic behavior of the vibration generator in terms of its operation efficiency.

## 4. Analysis of the System Effectiveness

**Figure 6:**
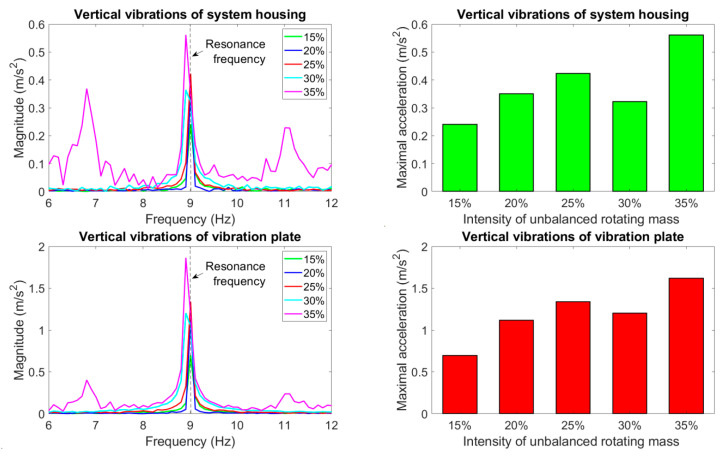

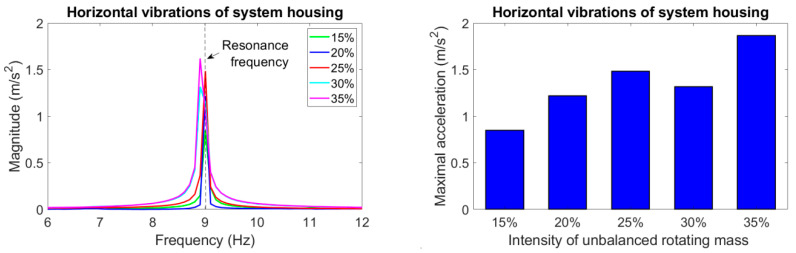
The amplitude spectra of the vibration acceleration of the generator housing and the vibration plate measured in the vertical and horizontal directions using different values of the intensity of unbalanced mass, as well as their corresponding maximum values of resonant vibrations.

The analysis of dynamic behavior was aimed at determining the maximum vibration amplitudes of the vibrating plate in relation to the lowest possible vibrations of the generator housing, taking different intensities of the unbalanced mass into account. In the analyzed load case (466 kg), we considered the resonant vibrations of the mechanical system which occurred at the motor rotational speed of 540 rpm, corresponding to the frequency of system oscillations close to 9 Hz. The amplitude spectra of the vibration acceleration of the generator housing and the vibration plate measured in the vertical and horizontal directions at different values of the intensity of unbalanced mass are shown in Figure 6 on the left. In Figure 6 on the right, the corresponding maximum values of the resonant vibration amplitudes are presented.

According to the test results presented in Figure 6, increasing the intensity of unbalanced mass of the motor usually resulted in higher amplitudes of vibrations measured on the vibrating plate connected to the suspension system of galvanized elements. However, the generated vibrations were also transferred to the system housing in the vertical and horizontal directions. An initial growth of the maximum acceleration was observed due to the increased intensity of the unbalanced rotating mass (up to 25%). A significant decrease in the vibration amplitudes of the system housing was obtained for an unbalance intensity equal to 30%. Under these operating conditions, the best system efficiency was achieved, for which a substantial amplification of vibration amplitudes was attained on the vibrating plate in relation to the system housing. In the case this unbalance intensity, both the vertical and horizontal vibrations of the generator housing showed acceptable values of the measured acceleration amplitude (in Figure 6, right). Although further increasing the unbalanced mass (up to the intensity of 35%) again led to higher amplitudes of the vibrating platform, this nonlinear increase resulted in the appearance of many natural frequencies of the frame structure used to mount the generator. As a result, vibrations were generated in a wide range of frequencies (Figure 6, left), which contributed to significant disturbances during the operation of the tested generator.

**Figure 7:**
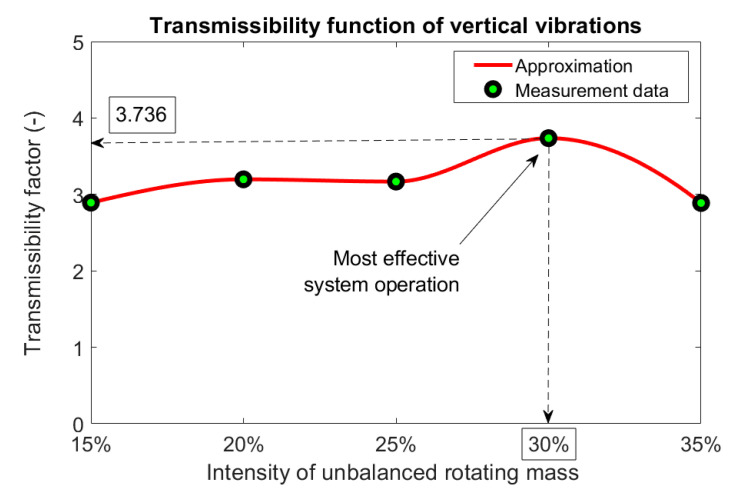
Transmissibility function of the vertical vibration during the system’s operation near its resonance frequency.

In order to determine the highest efficiency of the generator’s operation, the vibration transmission function was developed as a ratio of the maximum values of the vibration amplitude measured on the vibrating plate in relation to the maximum values of the vibration amplitude measured on the generator housing (Figure 7). This ratio was determined for vertical vibrations generated during the system’s operation near its resonance frequency (about 9 Hz), which corresponds to a motor rotational speed of 540 rpm.

As it resulted from the shape of a nonlinear function (Figure 7), the transmissibility factor maintained a relatively constant value (around 3) for most of the intensities considered. The only exception to this rule was an increased system effectiveness for the intensity of unbalanced mass equal to 30%. In this case, the greatest amplification of the resonant vibrations was noticed due to proper adjustment of the centrifugal force to the measured system dynamic properties. With this unbalance of the rotating mass, the amplitude of vibrations measured in the vertical direction on the vibration plate connected to the suspension system of galvanized elements was almost four times greater than the vibration amplitude of the generator housing. During the operation of the generator with this unbalanced mass, no excessive vibrations of its housing were observed in either vertical or horizontal directions. Moreover, the frame structure for mounting the generator did not show excessive oscillations around the static equilibrium position of the system.

## 5. Conclusions

The aim of this research was to develop vibration generators that can be used in the hot-dip galvanizing process. In the case of galvanizing elements of complex shape, it is not possible to use air knives to remove excess zinc from elements removed from the zinc bath. The developed method allows the use of vibration generators placed between the gantry and the platform with galvanized elements. Vibration generators ensure that the platform vibrates cyclically as galvanized elements are lifted from the zinc bath.

As part of the research work, the model of the mechanical vibration generator was verified under various operating conditions. A satisfactory agreement of the results of simulation and experimental tests was demonstrated for the motor rotational speed of 540 rpm and in the case of different unbalanced rotating mass intensities. The analysis of dynamic behavior clearly showed that the greatest efficiency of the proposed vibration generator was achieved near the resonance frequency of the system (about 9 Hz) and at the unbalance intensity of 30%. Under these operating conditions, an almost 4-fold increase in the vertical vibrations of the vibrating plate was obtained.

Correct operation of the vibration generator loaded with given masses, confirmed by tests, allows its use in further stages of tests involving the use of a vibration generator system (preferably two) placed between the gantry and the platform with galvanized elements.

## Data Availability

Data sharing is not applicable to this article.

## Appendix A. Numerical Parameters of the Simulation Model

Mass of the vibration plate with electrovibrator (*m_1_*)	144 kg
Mass of the element excited to mechanical vibration (*m_2_*)	466 kg
Mass of the system housing (*m_3_*)	434 kg
Stiffness of the lower springs (*c_1_*)	1254 N/mm
Stiffness of the suspension system (*c_2_*)	964,000 N/mm
Stiffness of the attachment in the vertical direction (*c_4_*)	2800 N/mm
Stiffness of the attachment in the horizontal direction (*c_5_*)	400 N/mm
Damping of the attachment in the vertical direction (*d_4_*)	20,000 Ns/m
Damping of the attachment in the horizontal direction (*d_5_*)	3000/m
Mass moment of inertia of the rotor with load (*J_e_*)	0.1 kgm^2^
Viscous friction coefficient (*d_e_*)	0.001 Ns/m
Total mass of the rotating element (*m_e_*)	20 kg
Lever arm of the rotating element (*r_e_*)	0.05 m
Number of pole pairs of the motor (*p*)	2
Stator leakage inductance (*L_ls_*)	0.009121 H
Rotor leakage inductance (*L_lr_*)	0.009695 H
Mutual inductance (*L_m_*)	0.2435 H
Stator resistance (*R_s_*)	8.431 Ω
Rotor resistance (*R_r_*)	8.856 Ω
